# Metformin as a potential agent for modulating the faulty endometriotic mesenchymal stem cells: A case-control study

**DOI:** 10.18502/ijrm.v20i10.12270

**Published:** 2022-11-02

**Authors:** Parisa Mashayekhi, Mehrdad Noruzinia, Sepideh Khodaverdi

**Affiliations:** ^1^Biotechnology Research Center, Pasteur Institute of Iran, Tehran, Iran.; ^2^Department of Medical Genetics, Faculty of Medical Sciences, Tarbiat Modares University, Tehran, Iran.; ^3^Endometriosis Research Center, Iran University of Medical Sciences, Tehran, Iran.

**Keywords:** Endometriosis, Mesenchymal stem cells, Metformin.

## Abstract

**Background:**

According to stem cell theory, it seems that the proliferation/differentiation imbalance in endometrial mesenchymal stem cells (enMSCs) is the leading cause of endometriosis, so targeting them to modulate stemness-relevant factors seems to be a wise choice for endometriosis treatment.

**Objective:**

We aimed to investigate the effects of metformin on stemness properties of enMSCs by evaluating the expression profile of stemness-related genes and microRNAs (miRNAs).

**Materials and Methods:**

In this case-control study, MSCs were isolated from the eutopic endometrium of 3 endometriotic and 3 healthy women. After their characterization and culture, they were treated with 0.1, 1, and 10 mM metformin for 72 hr. Finally, the expression of octamer-binding transcription factor (OCT) 4A, OCT4B, OCT4B1, sex determining region Y-Box transcription factor 2, nanog homeobox, microRNA-200b, microRNA-145, and lethal-7b were analyzed by quantitative reverse transcription-polymerase chain reaction.

**Results:**

Metformin modulated the expression of stemness-related genes and miRNAs, OCT4A, OCT4B, OCT4B1, sex determining region Y-Box transcription factor 2, nanog homeobox, microRNA-200b, microRNA-145, and lethal-7b in enMSCs, especially at 1 and 10 mM concentration. Notably, metformin had a paradoxical effect on normal enMSCs.

**Conclusion:**

We showed that metformin could modulate the expression of deregulated genes and miRNAs in faulty enMSCs, and restore their skewed self-renewal/differentiation balance, so it might be a promising drug for endometriosis treatment. The paradoxical effect of metformin on enMSCs and normal enMSCs might be because of their different metabolic patterns, so it requires further investigation to illustrate.

## 1. Introduction

Endometriosis is a benign debilitating gynecologic disorder with a growing prevalence and affects approximately 10% of reproductive-aged women and 50% of infertile women (1).

There are several theories about the cause of endometriosis, and stem cell theory is the most contentious. This theory states that stem cells play a key role in endometriosis development. Since retrograde menstruation has an important role in carrying the endometrial stem cells to the peritoneum; it also plays a crucial role in endometriosis development (2). Although retrograde menstruation frequently occurs in reproductive-aged women, endometriosis occurs in only 10%.

As a result, it seems that endometriotic mesenchymal stem cells (enMSCs) are different from normal stem cells: they have shorter doubling time, high proliferation activity, decreased decidual response (3, 4), and it also appears that they have skewed proliferation/differentiation balance. The pluripotency regulated transcription factors (octamer-binding transcription factor [OCT] 4, sex determining region Y-Box transcription factor 2 [SOX2], and nanog homeobox [NANOG]) (5), in cooperation with epigenetic regulatory modulators like microRNAs (miRNAs), fine tunes the self-renewal/ differentiation balance in stem cells (6). Indeed, increased expression of these transcripts enhances the self-renewal, proliferation, and motility of stem cells despite decreases their differentiation (7). Transcriptional analyses and in vitro assays help identify the skewed expression of stemness-related genes and miRNAs in eutopic and ectopic endometrium of endometriotic women (8-10).

In our earlier studies, we also showed the skwed expression of OCT4A, OCT4B, OCT4B1, NANOG, miR-200b, let-7b, and miR-145 in enMSCs: they are involved in pathways that regulate self-renewal/differentiation balance in stem cells (11, 12).

Endometriosis treatment is admittedly a big challenge, and currently, there is no cure for endometriosis because of its unknown etiology. Considering the results of previous studies about the effect of metformin on the expression of stemness-related genes and miRNAs, we investigated the effect of metformin on enMSCs in this study.

Metformin controls endometriotic lesions by inhibiting inflammation-related cytokines, local estrogen productions, and eutopic stromal cell proliferation (13). Although there is no direct evidence of the effect of metformin on endometrial stem cells, several studies on cancers have shown that metformin reduces the expression of genes and microarrays that promote self-renewal. For example, metformin downregulates the expression of stemness markers such as CD44, NANOG, OCT-4, c-Myc (14-16), and also reprogrammes cancer stem cells (17). Furthermore, metformin promotes MSCs differentiation via AMPK activation. For example, metformin modulates the expression of miRNAs in several types of cancer, like pancreatic cancer, esophageal squamous carcinoma, gastric cancer, and prostate cancer (18).

Accordingly, metformin might be an effective endometriosis treatment by correcting the disturbed balance in enMSCs. In this study, we mainly focused on the effect of metformin on the expression of stemness-related genes (OCT4A, OCT4B, OCT4B1, SOX2, and NANOG) and miRNAs (miR-200b, miR-145, and let-7b) to examine whether metformin affects the proliferation/differentiation balance in enMSCs.

## 2. Materials and Methods

### Specimen sources

In this case-control study, human endometrial tissue was obtained from premenopausal women aged 30-45 yr (mean 34.8 ± 4.7) in the secretory phase. We recruited 3 healthy fertile women as the control group and another 3 with stage III and IV endometriosis who went under laparoscopy at the Rasoul Akram hospital of Iran Medical University, Tehran, Iran. Women with endometrial anomalies like polyps, hyperplasia, or cancer, besides those who take hormonal treatment and gonadotropin-releasing hormone agonists, were excluded from the study.

### Isolation and culture of human endometriotic mesenchymal stem cells

The endometrium layer was separated from the myometrium and washed in phosphate-buffered saline (PBS), then minced into 1-2 mm^3^ pieces in a medium containing Dulbecco modified Eagle medium. Ham's F-12 (DMEM.F-12; Invitrogen, UK) and 1% penicillin-streptomycin antibiotic solution (Invitrogen, USA) were used. Enzymatic digestion was done with collagenase type 3 (300 µg/ml; Sigma, Germany) at 37 C for 90 min to obtain the cell suspensions. The cell suspension was filtered through 150, 100, 40 mm wire sieve to remove undigested tissue and epithelial components. Endometrial stromal cells were then cultured in T25 culture flasks containing DMEM/F-12 (Invitrogen, UK), 1% penicillin-streptomycin solution, and 10% fetal bovine serum (FBS, Gibco, USA) (19).

### Endometrial stromal cells flow cytometry analysis 

Isolated stromal cells were trypsinized and centrifuged. Cell pellets were resuspended in PBS containing 5% FBS and incubated for 45 min on ice then centrifuged. Cell pellets were resuspended in PBS containing 5% FBS and incubated with PE or FITC-conjugated antibodies for 30 min at 4 C in the dark. Hematopoietic stem cells specific antibodies served as negative controls (FITC-conjugated anti-human CD45 [BD Bioscience, USA] and CD34 [IMMUNOSTEP, Spain]), and PE-conjugated anti-human CD90, CD105, CD73, and CD146 (BD Bioscience, USA) were used as MSCs specific antibodies. Fluorescence-activated cell sorting (FACS) was done on the FACS Calibur apparatus (Becton Dickinson, USA), while data analysis was done with the FlowJo 7.6 software.

### Differentiation of enMSCs

enMSCs (CD146+, CD90+, CD105+, CD73+, and CD34-, CD45-) were seeded in 24-well plates and cultured in differentiation media for 4 wk, then they were fixed and stained with 4% Alizarin Red (pH 4.1) and 1% Oil Red (Sigma, Germany) respectively to assess their differentiation into osteogenic and adipogenic lineages.

### Cell viability assay

We used the 3-(4,5-dimethylthiazol-2-yr)-2,5-diphenyltetrazolium bromide (MTT) test to evaluate the metabolic activity, proliferation, and viability of alive cells; we cultured the endometrial MSCs (150 µL; 2.5 × 104 cells per well) in 96-well microplates containing 10% FBS, 24 hr later, enMSCs were treated with serial dilutions of metformin (0.1, 1, and 10 mM (Sigma-Aldrich), and then they were incubated for 72 hr at 37 C and 5% CO ${}_{2}$ . After 72 hr, we washed cells and incubated them with 0.5 mg/mL of 3-(4,5-dimethylthiazol-2-yr)-2,5-diphenyl tetrazolium bromide (MTT) at 37 C for 4 hr, then we throw away the medium and added dimethyl sulfoxide to solubilize the formazan crystals. Then the absorbance for each well was measured at a wavelength of 490 nm using an ELISA reader. Independent experiments were repeated in triplicate.

### RNA extraction and cDNA synthesis

We used TRIzol (Sigma Germany) to extracte the cellular RNA, then evaluated the quality and quantity of extracted RNA using nanodrop (the ratio of absorbance at 260 and 280 nm ≥ 1.8) and denaturing agarose gel electrophoresis. For cDNA synthesis, we used 0.5-1 μg of total RNA with random hexamer primers for genes (OCT4A, OCT4B, OCT4B1, SOX2, NANOG, and GAPDH) and specific stem-loop primer for miRNAs (20) (miR-200b, let-7b, miR-145, and RNU44) in a 20 μl reverse-transcriptase reaction mixture (Takara Bio, Japan).

### Real-time PCR for mRNAs and miRNAs expression detection

To determine the expression of OCT4A, OCT4B, OCT4B1, SOX2, NANOG, miR-200b, miR-145, and let-7b, we used the SYBR Green Assay kit (Applied Biosystems, UK), following the manufacturer protocol. Sequences of PCR primers were presented in our earlier studies (11, 12). We accomplished real-time PCR reactions in 10 μL of the reaction mixture by AB StepOne real-time PCR System (Applied Biosystems, UK), followed by analyzing the data by the Pfaffl method. *GAPDH* and *RNU44 *were used to normalize the expression value of genes and miRNAs*,* respectively.

### Ethical considerations

Ethics approval for this study was obtained by the Ethics Committee of Medical Faculty of Tarbiat Modares University, Tehran, Iran (No. 1395.409). Written informed consent was obtained from each participant.

### Statistical analysis

Statistical significance of variances between group means was analyzed using either Student *t* test or ANOVA using GraphPad Prism version 6.0.0 for Windows, GraphPad Software, San Diego, California USA. Results considered significant at a p < 0.05.

## 3. Results

### Isolation and characterization of enMSCs

**Figure 1:**
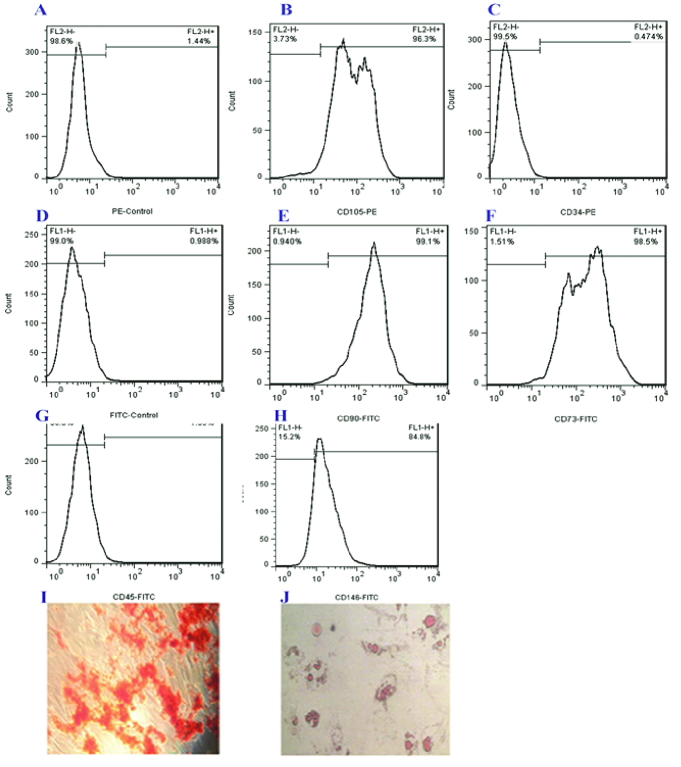
Characterization of endometrial MSCs. Human endometrial MSCs positively expressed (A) PE-conjugated isotype control, (B) CD105 (96.3%), (C) CD34 (0.474%), (D) FITC-conjugated isotype control, (E) CD90 (99.1%), (F) CD73 (98.5%), (G) CD45 (1.99%), and (H) CD146 (84.8%). They also exhibited (I) Adipogenic, (J) Osteogenic differentiation potential.

MSCs were isolated from the endometrium and cultured, then the expression of mesenchymal and hematopoietic markers was assessed. Flow cytometer analysis confirmed the expression of mesenchymal markers CD73 (98.5%), CD90 (99.1%), CD105 (96.3%), and CD146 (84.8%). The expression of hematopoietic markers including CD45 (1.99%) and CD34 (0.474%) was negative in isolated cells (Figure 1A-H). We induced cells with specific differentiation media to evaluate their adipogenic and osteogenic differentiation potential and then visualized them by staining the lipid vacuoles and calcium deposits with oil red and alizarin red, respectively (Figure 1I and J).

### Metformin does not influence cell viability up to 10 mM

**Figure 2:**
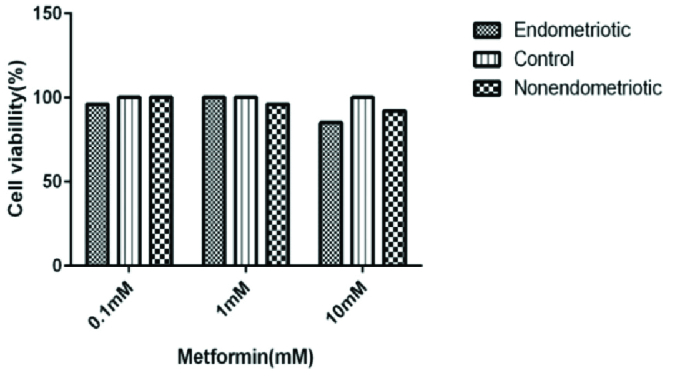
MSCs were plated in 96 well plates and either treated or not treated with 0.1, 1, and 10 mM metformin for 72 hr, followed by MTT test. Values are shown as living cells percentage relative to the control untreated cells which were set at 100% in control values. Results expressed the Mean ± SD (n = 3).

To evaluate the different doses of metformin on cell viability, we incubated MSCs with 0.1, 1, and 10 mM metformin for 72 hr. Then we conduct an MTT assay to assess mitochondrial activity. Cell viability was over 80% in treated cells in comparison with the untreated group (Figure 2).

### Metformin mediated downregulation of *OCT4* transcripts (OCT4A, OCT4B, and OCT4B1) 

**Figure 3:**
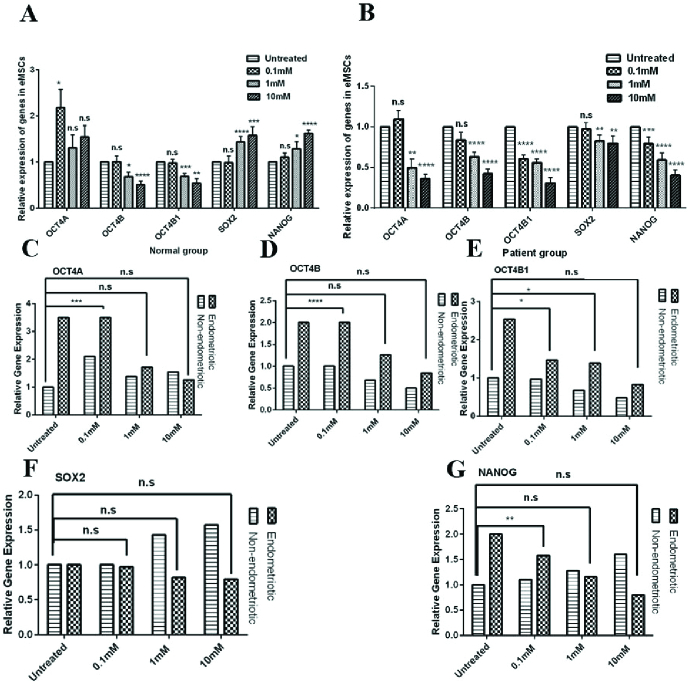
OCT4A, OCT4B, OCT4B1, SOX2, and NANOG relative expression following the metformin treatment in endometrial mesenchymal stem cells of 3 affected and 3 normal individuals, evaluated by quantitative real-time polymerase chain reaction (RT-PCR) (A-B). Comparative expression of stemness-related genes between enMSCs and untreated-normal enMSCs after treatment with metformin (C-G). mM: Millimolar, n.s: Nonsignificant. Variance between group means was analyzed using either Student *t* test or ANOVA using GraphPad Prism version 6.0.0. (*p < 0.05, **p ≤ 0.01, ***p ≤ 0.001, and ****p ≤ 0.0001).

To evaluate the effect of different doses of metformin on OCT4 transcripts, we incubated isolated normal MSCs and enMSCs with 0.1 mM, 1 mM, 10 mM metformin for 72 hr. Expression of OCT4A, OCT4B, and OCT4B1 with the different doses of metformin was shown in figures 3A and 3B and table I. In normal enMSCs, the 72 hr treatment with 1 and 10 mM metformin diminished the expression of OCT4B and OCT4B1 significantly in comparison to untreated MSCs. OCT4A expression showed a significant reduction with 0.1 mM metformin (Figure 3A), (Table I) treatment.

After the 72 hr treatment of enMSCs with metformin, it was observed that there is a significant reduction in the expression of OCT4 transcripts at all concentrations, especially with 1 and 10 mM doses (Figure 3B), (Table I). To evaluate the modulating effect of metformin on deregulated OCT4 transcripts in enMSCs, we compared the expression patterns between enMSCs and untreated-normal enMSCs; our results showed that with 1 and 10 mM metformin, the expression levels of OCT4A (p = 0.34 and p > 0.99) and OCT4B (p = 0.2 and p = 0.6) in enMSCs were not significantly different from those of the untreated-normal enMSCs, indicating its corrective effect on enMSCs (Figure 3C, D). Also, OCT4B1 expression in enMSCs at 10 mM metformin concentration was not significantly different from that of untreated-normal enMSCs (p = 0.79) (Figure 3E).

### Metformin treatment repressed the gene expression of *SOX2* and *NANOG* in a dose-dependent manner

Seventy-two hr treatment of normal enMSCs with 1 and 10 mM metformin compared to untreated MSCs showed significantly increased expression of *SOX2* and *NANOG* (Figure 3A) (Table I). After the 72 hr treatment of enMSCs with metformin, expression of *SOX2 *and* NANOG* reduced significantly at all metformin concentrations for NANOG and 1 and 10 mM for *SOX2* compared with untreated-enMSCs (Figure 3B) (Table I). Comparing the expression levels of *SOX2 *between enMSCs and untreated-normal enMSCs showed that all metformin concentrations had modulating effect on *SOX2* expression (Figure 3F). NANOG expression in enMSCs treated with 1 and 10 mM metformin was not significantly different from the value of untreated-normal enMSCs (p > 0.99 and p = 0.34). Indicating that metformin had a modulator effect on NANOG expression in enMSCs (Figure 3G).

### Metformin mediates downregulation of *miR-200b* in enMSCs

**Figure 4:**
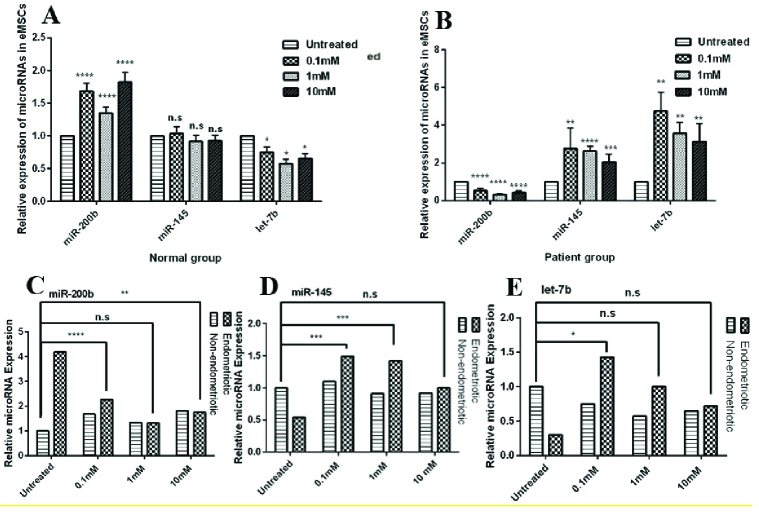
Relative expression of *miR-200b, miR-145, and let-7b* after the treatment of endometriotic mesenchymal stem cells and normal endometrial mesenchymal stem cells with metformin, evaluated by quantitative real-time polymerase chain reaction (RT-PCR) (A and B). Comparative expression of stemness-related miRNAs between endometriotic MSCs and untreated-normal endometrial MSCs after treatment with metformin (C-E). mM: Millimolar, n.s: Nonsignificant. Variances between group means was analyzed using either Student *t* test or ANOVA using GraphPad Prism version 6.0.0. (*p < 0.05, **p ≤ 0.01, ***p ≤ 0.001, and ****p ≤ 0.0001).

In normal enMSCs, all metformin concentrations had an increasing effect on miR-200b expression after 72 hr compared to untreated-enMSCs (Figure 4A) (Table II). After the 72 hr treatment of enMSCs, there was a significant reduction of miR-200b expression at all concentrations compared to untreated-enMSCs, (Figure 4B) (Table II). Comparing the expression level of miR-200b between enMSCs and untreated-normal enMSCs (p = 0.6) showed that with 1 mM metformin, the expression levels were not significantly different between the 2 mentioned groups, so in that concentration, metformin had a modulating effect on faulty enMSCs (Figure 4C).

### Metformin up-regulates the expression of *miR-145* and *let-7b* in enMSCs

In normal enMSCs, all metformin concentrations decreased the let-7b expression compared to untreated-enMSCs, but the miR-145 expression was not influenced by any metformin doses (Figure 4A) (Table II). The results showed that after 72 hr treatment with metformin, the expression of miR-145 and let-7b in enMSCs had decreased significantly at all concentrations compared to untreated-enMSCs (Figure 4B) (Table II). miR-145 expression level in enMSCs treated with 10 mM metformin was similar to that of untreated-normal enMSCs (p > 0.99) (Figure 4D). let-7b expression levels in enMSCs treated with 1 and 10 mM metformin increased and were not significantly different from untreated-normal enMSCs (p > 0.99 and p = 0.61) (Figure 4E).

**Table 1:** Summary of metformin effect on the expression of OCT4A, OCT4B, OCT4B1, SOX2, and NANOG in endometrial MSCs of endometriotic and normal individuals after treatment with 0.1 mM, 1 mM, and 10 mM metformin


	**Endometriotic MSCs**			**Normal MSCs**		
	**0.1 mM**	**1 mM**	**10 mM**	**0.1 mM**	**1 mM**	**10 mM**
*OCT4A*	1.09 ± 0.1 (p = 0.3)	0.49 ± 0.1 (p = 0.001)	0.36 ± 0.08 (p < 0.0001)	2.1 ± 0.3 (p = 0.01)	1.3 ± 0.2 (p = 0.3)	1.5 ± 0.2 (p = 0.05)
*OCT4B*	0.83 ± 0.21 (p = 0.1)	0.63 ± 0.05 (p < 0.0001)	0.42 ± 0.05 (p < 0.0001)	1 ± 0.1 (p = 0.97)	0.68 ± 0.1 (p = 0.01)	0.5 ± 0.08 (p = 0.0001)
*OCT4B1*	0.59 ± 0.06 (p < 0.0001)	0.55 ± 0.04 (p < 0.0001)	0.3 ± 0.04 (p < 0.0001)	0.97 ± 0.07 (p = 0.7)	0.68 ± 0.07 (p = 0.0003)	0.53 ± 0.1 (p = 0.001)
*SOX2*	0.97 ± 0.05 (p = 0.6)	0.82 ± 0.05 (p = 0.002)	0.62 ± 0.07 (p = 0.0001)	0.98 ± 0.1 (p = 0.8)	1.43 ± 0.07 (p < 0.0001)	1.57 ± 0.1 (p = 0.0002)
*NANOG*	0.79 ± 0.05 (p = 0.001)	0.58 ± 0.06 (p < 0.0001)	0.4 ± 0.04 (p < 0.0001)	1.1 ± 0.06 (p = 0.09)	1.28 ± 0.1 (p = 0.01)	1.6 ± 0.05 (p < 0.0001)
The results are presented as the fold change relative to 0 hr. Data were presented as the Mean ± standard deviation, analyzed using one-way analysis of variance and then compared among groups using Student's *t* test. A significance threshold of p < 0.05 was used						

**Table 2:** Summary of metformin effect on expression of miR-200b, miR-145 and let-7b in enMSCs and normal enMSCs after treatment with 0.1 mM, 1 mM, and 10 mM metformin


	**Endometriotic MSCs**			**Normal MSCs**		
	**0.1 mM**	**1 mM**	**10 mM**	**0.1 mM**	**1 mM**	**10 mM**
*miR-200b*	0.51 ± 0.03 (p < 0.0001)	0.31 ± 0. 02 (p < 0.0001)	0.42 ± 0.02 (p < 0.0001)	1.68 ± 0.08 (p < 0.0001)	1.34 ± 0.06 (p < 0.0001)	1.82 ± 0.09 (p < 0.0001)
*miR-145*	2.7 ± 0.4 (p = 0.002)	2.63 ± 0.1 (p < 0.0001)	2 ± 0.1 (p = 0.0001)	1.1 ± 0.2 (p = 0.6)	0.9 ± 0.2 (p = 0.6)	0.9 ± 0.2 (p = 0.7)
*Let-7b*	4.57 ± 0.99 (p = 0.002)	3.56 ± 0.57 (p = 0.002)	3.1 ± 0.94 (p = 0.002)	0.75 ± 0.3 (p < 0.04)	0.75 ± 0.3 (p < 0.01)	0.65 ± 0.31 (p = 0.04)
The results are presented as the fold change relative to 0 hr. Data were presented as the Mean ± standard deviation, analyzed by one-way analysis of variance and then compared among groups using Student's *t* test. A significance threshold of p < 0.05 was used						

## 4. Discussion

Stem cell theory is a controversial issue which posits that the cells responsible for the regeneration of the endometrial lining play a role in the development of endometriosis. Several studies showed that enMSCs have different characteristics and functions. Although they increase self-renewal, proliferation, and migration (4), they have decrease differentiation (3), resulting in the disrupted self-renewal/differentiation balance. Therefore, targeting the MSCs to restore the disrupted balance can be concluded as an effective strategy for endometriosis treatment. It is important because current therapeutic modalities not only fail to prevent high recurrence of endometriosis but also cause serious side effects. In this regard, Taghizadeh et al. showed that lovastatin treatment could increase the differentiation of enMSCs and decrease their stemness properties (21).

In this study, we investigated the effect of metformin on disturbed self-renewal/differentiation equilibrium in enMSCs by evaluating the impact of different doses of metformin on the expression of OCT4A, OCT4B, OCT4B1, SOX2, NANOG, miR-200b, miR-145, and let-7b in enMSCs. These genes and miRNAs are involved in the determination of stem cell fate, and also in our previous transcriptional study, they had skewed expression in enMSCs (11, 12). Treatment with 1 and 10 mM metformin reduced the elevated levels of OCT4A, B, B1, and NANOG in enMSCs to the normal level, the elevated expression level of *miR-200b* decreased with 1 mM metformin, while the reduced level of *miR-145* and *let-7b* increased with 10 mM and 1 mM metformin, approaching normal expression value.

Our findings were consistent with experiments were done on cancer stem cells. For example, metformin inhibited self-renewal, proliferation, migration, and invasion by decreasing the expression of CSC markers (22). In ovarian and breast cancers, metformin causes reprogramming in CSCs and converts them into non-CSCs (14). Similarly, metformin causes reexpression of miRNAs (let-7a, let-7b, miR-26a, miR-101, miR-200b, and miR-200c) and attenuates CSC function by decreasing the expression of CSC markers in pancreatic cancer (23). Besides, metformin up-regulates the *miR-145* and downregulates the Wnt. β-catenin signaling pathway in colon cancer stem cells (24). In breast cancer, epithelial cell treatment with 10 mM metformin up-regulates *let-7* expression, thus retains the differentiated state of mammary epithelium epigenetically and inhibited EMT-related self-renewal of cancer developing cells (25). Furthermore, metformin causes differentiation in bone marrow progenitor cells both in vivo and in vitro (17). Using metformin to modulate the expression of deregulated genes and miRNAs in enMSCs, we showed that the skewed self-renewal/differentiation balance in enMSCs is reversible. So, it is reasonable that metformin could also directly affects the enMSCs in vivo.

Considering the safety and low side effect profile of metformin, besides its correcting impact on disrupted self-renewal/differentiation balance, it might be a promising drug for endometriosis treatment. According to our results, metformin had a paradoxical effect on normal enMSCs in comparison to enMSCs. Its underlying mechanisms were reviewed the previous studies. We realized that endometriotic lesions were similar to cancers; they grew uncontrollably and invasively, and they had increased new blood vessels and decreased cellular apoptosis. Considering the fact that metformin has significant impacts on mitochondrial metabolism by decreasing oxidative phosphorylation and increasing anaerobic glycolysis (26) and given its contradictory effect on normal enMSCs compared to enMSCs, it could indirectly implicate that endometriotic MSCs are like cancer stem cells (27), favor oxidative phosphorylation to generate energy, and different from normal stem cells that use anaerobic glycolysis. Consequently, the paradoxical impact of metformin on enMSCs in comparison to normal endometrial MSCs could be the result of their different cellular metabolism.

## 5. Conclusion

In the current study, we showed that metformin could modulate the expression of deregulated genes and miRNAs in enMSCs, and may correct their skewed self-renewal/differentiation balance. It might be a promising drug for endometriosis treatment.

Furthermore, the hypothesis of different metabolic patterns in normal MSCs and enMSCs could provide a clear explanation for different characteristics of enMSCs and their paradoxical response to metformin compared to normal MSCs. Our results are an important source of information about the in vitro effect of metformin on enMSCs.

##  Conflict of Interest

The authors declare that there is no conflict of interest.
